# Synergistic Communication between CD4+ T Cells and Monocytes Impacts the Cytokine Environment

**DOI:** 10.1038/srep34942

**Published:** 2016-10-10

**Authors:** Sarah B. Schrier, Abby S. Hill, Deborah Plana, Douglas A. Lauffenburger

**Affiliations:** 1 Department of Biological Engineering, Massachusetts Institute of Technology, Cambridge, MA 02139, USA

## Abstract

Physiological cytokine environments arise from factors produced by diverse cell types in coordinated concert. Understanding the contributions of each cell type in the context of cell-cell communication is important for effectively designing disease modifying interventions. Here, we present multi-plexed measurement of 48 cytokines from a coculture system of primary human CD4+ T cells and monocytes across a spectrum of stimuli and for a range of relative T cell/monocyte compositions, coupled with corresponding measurements from PBMCs and plasma from the same donors. Computational analysis of the resulting data-sets elucidated communication-independent and communication-dependent contributions, including both positive and negative synergies. We find that cytokines in cell supernatants were uncorrelated to those found in plasma. Additionally, as an example of positive synergy, production levels of CXCR3 cytokines IP-10 and MIG, depend non-linearly on both IFNγ and TNFα levels in cross-talk between T cells and monocytes. Overall, this work demonstrates that communication between cell types can significantly impact the consequent cytokine environment, emphasizing the value of mixed cell population studies.

Immune cells communicate with each other and with tissue cells to mount an effective response to pathogens or maintain homeostasis. Communication and activation of the immune cell network can occur by cell-cell contacts as well as by secretion of cytokines and chemokines. Many studies have demonstrated that cytokine profiles from human serum and plasma can reveal important information about disease state, including in pathologies such as cancer^1^,^2^,^3^ and autoimmune diseases^2^,^4^,^5^,^6^. However, low sensitivity of detection, as well as similarity of cytokine profiles between different disease states, have limited the clinical utility of cytokine profiling^7^,^8^.

Although it is possible to identify changes in circulating cytokines relevant to inflammation in serum samples, it is difficult to derive mechanistic information about overall change in immune activation from these measurements. To identify cytokines mediated by activation of immune cells, additional efforts have measured cytokine secretion from stimulated peripheral blood mononuclear cells (PBMCs) or whole blood. These cell-based measurements have been useful over the past several years in differentiating disease from health and predicting response to treatment^9^,^10^,^11^,^12^,^13^,^14^.

While measurements of cytokine secretion by PBMCs may lead to a more robust signature of disease, these measurements miss individual contributions of each cell type. As a result, gleaning information about secretion from each immune cell type from PBMC data alone requires further computational deconvolution or experimental analysis^15^,^16^,^17^. Interpretation of serum or plasma measurements is additionally complicated as the corresponding cells or tissues that lead to an observed cytokine may not be accessible. Consumption of cytokines or diffusion into tissue may lead to an underestimation of their secreted level. As such, there is a need for systems that can both identify cytokines that may be dysregulated in disease, as well as predict which immune cells are responsible for the observed pathology.

One complicating factor in directly interpreting cytokine secretion may be interactions between immune cells that change overall levels of observed cytokines. Interactions between immune cells, such as monocytes and CD4+ T cells, are implicated in the severity of several autoimmune diseases^1^,^2^,^3^,^18^,^19^. Additionally, autocrine and paracrine signaling, spatial effects, or sensitivity to cytokine levels even among cells of the same type can greatly impact cytokine secretion or other cellular behaviors^2^,^4^,^5^,^6^,^20^,^21^,^22^,^23^. Mechanistic models of communication between every contributing immune cell type, while ideal, would require a greater amount of sample than is generally available. As such, data-driven models have been shown to be useful for interpreting cell-cell communication and cell fate decisions in cytokine networks^7^,^8^,^24^,^25^,^26^,^27^,^28^.

Here, we present a comprehensive dataset of cytokine secretion measured from co-cultured primary human CD4+ T cells and monocytes under multiple stimulation conditions, as well as PBMC and plasma measurements from corresponding subjects. By measuring the individual contribution of each isolated cell type to responses elicited by several stimuli, we are able to directly identify cytokines and chemokines that are differentially secreted in the coculture environment of CD4+ T cells and monocytes. We suggest that common measurements of cytokine secretion may be missing important contributions from the multicellular environment, and additional measurements of cell-cell interactions are necessary to better understand how communication between cell types affects overall PBMC behavior.

## Results

### Coculture environment yields insights into divergent cellular behavior due to immune cell communication

**Figure 1:**
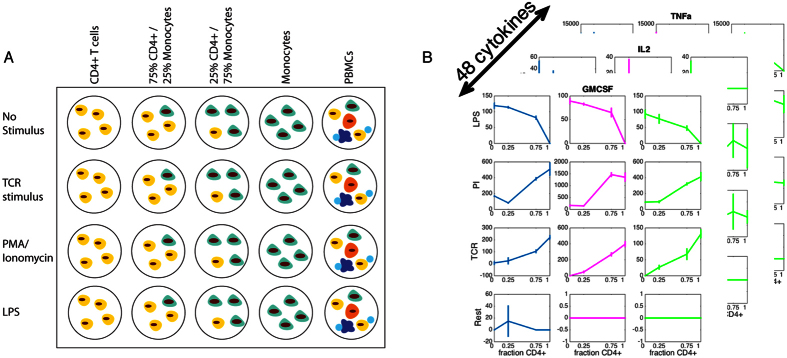
Study design to identify population specific cytokine secretion. (**A**) Conditions represented by this study. Cells were seeded as either isolated cell types or 25/75% mixtures of CD4+ T cells or monocytes as well as PBMCs isolated from each of three subjects. Total cell number was maintained across each well. Each sample was collected in technical triplicate, and for three donors, on the same day. Plasma from the same three donors was frozen at the time of cell isolation (not shown) (**B**) Representative data for each of the cytokines measured. Plots are of one of the measured 48 cytokines for three donors (columns) under four stimulation conditions (rows) across increasing CD4+ T cell fraction, where the remainder is made up of monocytes. Error bars represent standard deviations of technical triplicate measurements.

**Figure 2:**
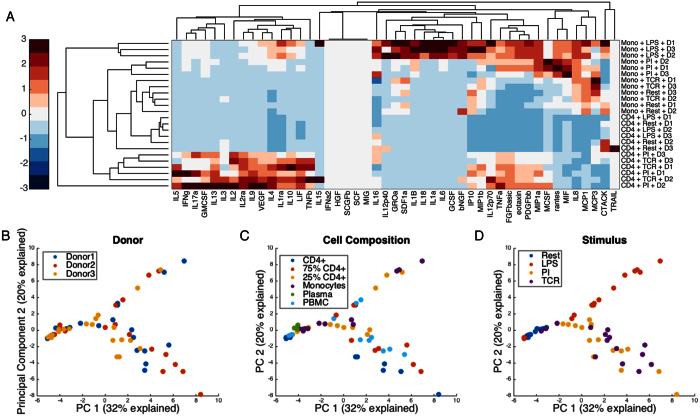
Both cell composition and stimulus are needed to interpret cytokine secretion. (**A**) Hierarchical clustering of all cytokines measured in isolated cell types across four stimuli. Data were mean centered and variance scaled along each column before clustering. (**B–D**) Principal components analysis of all data displayed along the first two principal components, with each point colored according to either donor (panel B) Cell composition or plasma (panel C) or stimulation (panel D).

To understand the behavior of communicating immune cells, we measured 48 cytokines and chemokines across three human donors (Fig. 1). Cells were plated alone or in combination as enriched CD4+ T cells, enriched monocytes, 75/25% mixtures, or seeded whole PBMCs at the same cell density, and left unstimulated or treated with one of three stimuli: lipopolysaccharide (LPS), phorbol 12-myristate 13-acetate/ionomycin (PI), and anti-CD3/CD28 coated micro-beads as a T-cell receptor stimulus (TCR). We simultaneously collected and measured cytokine concentrations in plasma from the same three donors (Supplementary Fig. S1). Cell separation was validated by staining for CD4 or CD14 (Supplementary Fig. S2). Viability was assessed by staining with propidium iodide using cells from one subject after 24 hours. While monocytes had a lower viability than CD4+ T cells, communication between the two cell types did not appear to affect viability (Supplementary Fig. S3). For each stimulation condition and cell type, we establish that isolated cell populations respond when stimulated with the relevant stimuli– we observe cytokines typically associated with T cells treated when adding TCR stimulus or PI to isolated CD4+ T cells (i.e. IL-2, IL-4, IL-13, GM-CSF, IFN-γ) and monocyte-specific cytokines when monocytes were treated with LPS (i.e. GRO-α, IL-1β, G-CSF). We applied hierarchical clustering to determine that CD4+ T cells stimulated with their relevant stimuli (PI and TCR) cluster separately from resting CD4+ T cells and those stimulated with LPS. Meanwhile, monocytes stimulated with LPS cluster separately from resting or TCR stimulus. We also note a low level of activation of monocytes by PI (Fig. 2A).

We performed principal components analysis on the full dataset to determine any natural separation in the data. Principal components analysis determines new axes in the data, made up of contributions from existing variables, that account for the most variance in the data. In this case, the first two components explain 53% of the variance in the data, and provide an intuitive two-dimensional visualization of the data. Figure 2B–D represents the same principal components analysis where each point represents one measurement (a single cell type/mixture condition under one stimulation condition, or plasma). The PCA plot demonstrates that the data does not separate cleanly by donor and that cell type or plasma alone also does not separate the data (Fig. 2B,C). Data does cluster by stimulus in the case of cells activated by their relevant stimuli: principal component (PC) one separates activated from resting cells (or plasma), and PC2 separates monocyte activation by LPS, positively loaded on PC2, from activated T cells, which are negatively loaded on PC2 (Fig. 2D). The PBMCs cluster together with the stimulated CD4+ T cells when stimulated with TCR/PI and with the monocytes when stimulated with LPS. This multidimensional dataset presents opportunities to examine relationships between commonly studied plasma and cell supernatants, as well as uncover cytokines regulated by cellular communication.

### Cytokines found in plasma are not well represented by *in vitro* immune cell secretion

As many studies have measured serum and plasma cytokines levels in a variety of human disease states, we first looked at cytokines that were captured by plasma measurements. Nineteen of the 48 assayed cytokines were detected in any of the plasma samples, as compared with 46 in the cultured cells.

**Figure 3:**
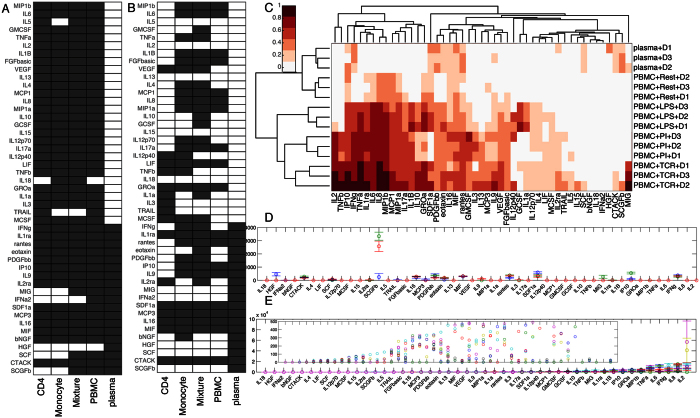
Cytokines present in plasma are not well represented by cell supernatants. (**A**) Cytokines present (black) or absent (white) under any stimulation or in plasma. (**B**) Cytokines secreted only in the resting case compared to those present in plasma. (**C**) Cytokines were rank ordered based on concentration in each sample, and samples were clustered based on that rank ordering. (**D**) Magnitudes of cytokines in plasma (top) or PBMCs (bottom) sorted by magnitude on the PBMC cytokine. Inset is a scaled up version of the PBMC data to show lower level secretion. Data are represented as mean ± standard deviation.

In an effort to determine which cells may be secreting cytokines characteristic of the plasma signature, we organized data by presence or absence of a cytokine under all conditions, for each category of cells (CD4+ T cells, monocytes, mixtures, combined across the two mixture fraction, PBMCs, or plasma) aggregated across the three donors. Three plasma cytokines (MIG, SCF, and SCGFb), are present in the mixtures and PBMCs, but not isolated cell types (Fig. 3A). One additional cytokine, IFNα2, is present only in PBMCs and plasma, and therefore may come from a cell type other than monocytes or CD4+ T cells in plasma. Only one cytokine, HGF, was detected in plasma but not in cultured cell supernatants under any stimuli tested (Fig. 3A).

As many of these cytokines were present only in stimulated cells, which may explain their absence in plasma, we further looked at only those cytokines secreted by each cell population under resting conditions. In this case, five cytokines (MIG, IFNα2, HGF, SCF, and SCGFb) are not represented by cellular supernatants (Fig. 3B). However, there are many cytokines secreted by these cells at rest that are not present in plasma measurements. This may be because the concentrations of these proteins in plasma are below the assay limit of detection, or possibly because they are consumed, degraded, or have diffused into tissues.

To understand quantitative relationships between plasma and immune cell supernatants, all of the cytokines in each condition were rank-ordered according to secreted concentration, and the rank ordered values were clustered (similar to a Spearman distance clustering)., as concentrations are not directly comparable between cultured cells and plasma. The rank ordering of plasma samples clusters separately from any of the PBMC samples, although closest with the resting cells (Fig. 3C). Thirteen cytokines were detected in resting PBMCs, of which nine were also detected in plasma measurements. Finally, we observed that the overall cytokine concentrations are not well correlated between plasma and PBMCs under any condition. The highest concentration observed in plasma is of SCGFb, which is the in the bottom half of rank-ordered cytokines secreted by PBMCs (Fig. 3D). The two most abundant cytokines in PBMCs, IL-2 and IL-8, are not observed in plasma in any of the three donors (Fig. 3E).

### PBMC secretion is better represented by mixtures than by isolated cell types

**Figure 4:**
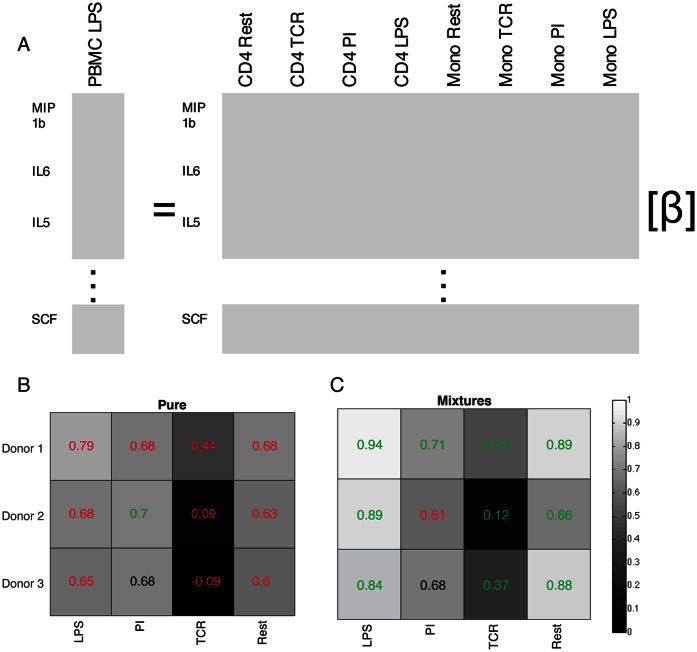
Mixtures are more predictive of the secretion of PBMCs than isolated cell types. (**A**) PBMC data for each stimulus (3 donors and 47 cytokines) were regressed against the corresponding data for each of the 8 conditions shown, and then separately for the same 8 conditions with the two concentrations of mixtures using linear regression. (**B**) Cross validation by leaving out all 47 cytokines for the donor shown was performed across all three donors on the models made of either isolated cell types or mixtures. R^2^ between the model and left out data is printed on each block. Green R^2^ are better fits using that data type, red are worse, and black is that the fit is the same whether using isolated cell types or mixtures.

PBMCs are commonly collected for functional studies of immune response. Additionally, significant effort has been put into isolating and characterizing the response of individual isolated immune cell subtypes. To better understand contributions of individual cell types or our mixture populations to PBMC secretion profiles, we performed eight linear regressions, separately for either isolated cell types or mixtures to PBMCs under each stimulus, as illustrated in Fig. 4A. Each PBMC condition was regressed against either the isolated cell types under all stimulation conditions or all of the mixture conditions. We chose to include all stimuli, instead of just the profiles under the same stimulus as the PBMC profile currently being predicted, as communication between cells in the PBMCs could cause secretion best resembling a single cell type under a different stimulation condition. This same rationale can also be applied to regressing mixtures to PBMCs, as not all cell types found in PBMCs were included in our cocultures.

As PBMCs are composed of CD4+ T cells and monocytes among other cell types, a linear model of these inputs plus an error term representing secretion from cells not accounted for should well explain the PBMC data if there is no communication. For a model built simultaneously for all three donors, across 47 cytokines and 4 stimuli to each of the PBMC stimuli, the R^2^ value for each regression can be found in Table 1.

We cross-validated our regression model by leaving out one full donor (all 47 cytokines under all 8 stimulation/cell types for both the mixture model and the isolated model). For each stimulus, for each donor left out, we applied the model built on the included data to the left out data. For 10 out of 12 cases (4 stimuli and 3 donors), the R^2^ value was greater for the model built on mixture data than individual cell types (Fig. 4B). For PBMCs treated with LPS or resting PBMCs, secretion appears to be especially well predicted by mixture conditions. PBMC stimulation with PI is approximately equally predictive for either the mixture cases or isolated cell types. Neither mixtures nor isolated cells provide an especially predictive fit for PBMCs stimulated with TCR stimulus – though mixtures are still more predictive than isolated cells. The discordance between this coculture system and PBMC secretion may be due to secretion from cell types not included in our model – in the case of TCR stimulus, this may be a result of CD8+ T cells in the PBMC population, which also respond to TCR stimulation. As the mixture models are more predictive of cytokine profiles from stimulated PBMCs, it seems as though collecting controlled mixture data is important in understanding what the overall network response of immune cells will be. Collecting cytokine secretion upon stimulation of isolated cell types is not sufficient to predict the cytokine environment in a multicellular environment such as that found in PBMCs; including interaction data improves the accuracy of the model.

### Cell-Cell Communication Impacts the Observed Cytokine Secretion Environment

**Figure 5:**
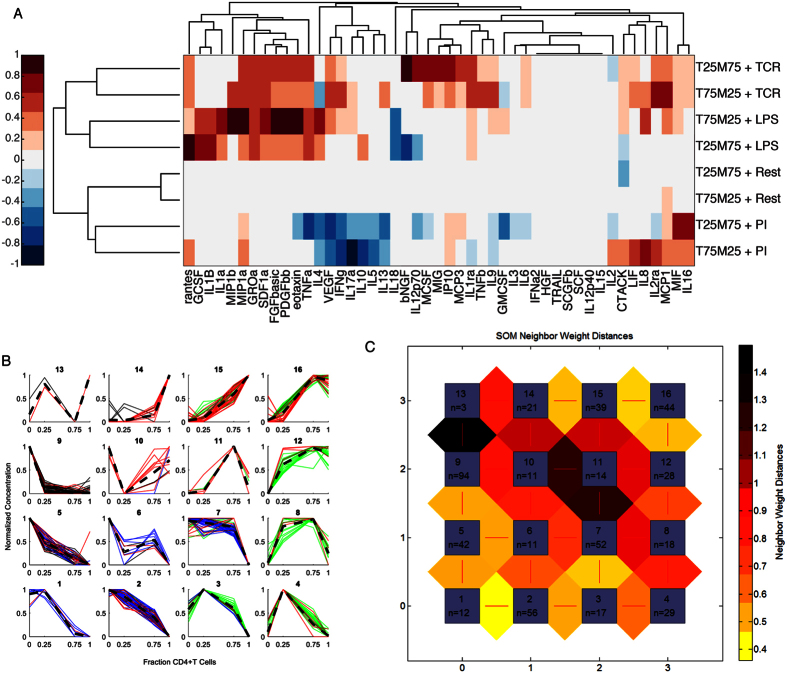
Most cytokines are significantly altered from their expected values in the presence of multiple cell types. (**A**) Differences between expected and observed values for each cytokine were calculated for each mixture fraction and stimulation condition. Differences were normalized to the maximum difference for each cytokine, and averaged across three donors only in the case where all three donors had the same sign for that condition. (**B**) Self-organizing maps organized cytokine profiles by primary cell source (CD4+ T cells, upper right; monocytes, lower left) and effect of communication (increase, lower right; decrease, upper left). (**C**) Distances between clusters show similarity between neighboring clusters.

To determine which cytokines are affected by communication between cell types, we can compare a value expected based on the individual populations to the value we observe when making that measurement experimentally. Here, we defined the expected value as the fractional sum of the concentration secreted by each cell type; for example, the “expected value” for TNFα secretion in the case of 25% monocytes stimulated with LPS would be 0.75*[TNFα]_CD4+ T,LPS_ + 0.25*[TNFα]_mono,LPS_ (Supplementary Fig. S4). To determine reproducibility, we considered only cytokines that had consistent upregulation or downregulation under each condition across all three donors (Fig. 5A). Using this metric, 40 out of the 47 measured cytokines were secreted differentially from the expected value under at least one of the four stimulation conditions. Two cytokines, CTACK and MCP-1, were consistently affected by the co-culture environment even in the resting condition, suggesting that either contact or soluble factor mediated feedback is present in these two cells types even in their unstimulated environments.

Several cytokines were highly increased in mixtures compared to isolated cell types, including IP-10, b-NGF, and M-CSF. G-CSF had a high mean fold-change, with the most-affected condition being 25% CD4+ T cells +75% monocytes under TCR stimulation. G-CSF is known to be produced in monocytes exposed to several cytokines produced by activated CD4+ T cells, including TNFα and IFNγ^9^,^10^,^11^,^12^,^13^,^14^,^29^. IL-18 was the cytokine most decreased due to communication in any donor, possibly due to a negative feedback loop by which multiple cytokines produced by activated CD4+ T cells and monocytes induce IL-18 binding protein^15^,^16^,^17^,^30^.

In addition to being strongly associated with particular cell types, sets of cytokines had different patterns of secretion with varying fractions of the two cell types. Self-organizing maps were used to identify these patterns of secretion resulting from CD4+ T cell – monocyte communication (Fig. 5B). We were interested in patterns that occur in any stimulation condition or in any individual donor, across any of the 47 cytokines detected above background, so data used consisted of a matrix of 564 cytokine-conditions by 4 fractional compositions.

Self-organizing maps are an unsupervised clustering technique that allows multi-dimensional data to be mapped into clusters in a lower-dimensional space, frequently in one or two dimensions to allow for visualization, where more similar clusters appear close together. For each cytokine and stimulation condition, the three donors were generally clustered into the same or neighboring clusters, with the median (0.667) of the mean distances between assigned clusters for the three donors for a given cytokine and stimulus resulting from two donors being in one cluster and the third being in a neighboring cluster. Only four conditions had a mean donor-to-donor cluster distance of greater than 2, two of which were resting (MCP-3 and SCGF-b), indicating possible differences in baseline activation of each cell population across subjects.

The algorithm identified a natural organization of the data by clustering together cytokines secreted primarily by monocytes (Fig. 5B, cluster 1) or by CD4+ T cells (Fig. 5B, cluster 16) as well as cytokines enhanced (Fig. 5B, cluster 4) or depleted (Fig. 5B, cluster 13) due to communication between the two cell types. As expected, 10 out of 12 conditions in cluster 1 were stimulated with LPS (blue), inducing strong monocyte activation, and all conditions in cluster 16 were stimulated with either TCR (green) or PI (red). While few cytokines were observed to decrease due to interactions between the cell types, many cytokines were increased. Of particular interest, cluster 4 included measures of IP-10, MIG, and IL-16 from all three subjects, indicating a consistently elevated response due to interactions between the cell types. Cluster 8 also included cytokines elevated in both mixture conditions relative to individual populations, including TCR-stimulated IL-1b and MIP-1b from all three donors. In the case of IL-1β, TCR stimulation of CD4+ T cells induces IFNγ, which in turn induces monocyte secretion of IL-1β^31^. IL-1β and MIP-1β are also known to induce secretion of each other in multiple cell types, suggesting positive feedback regulation among cytokines in this cluster^32^,^33^,^34^. IFNγ, a possible effector of these and other observed communication events, appears in clusters 9 and 7 in the absence of T cell stimulation and clusters 12, 14, 15, and 16 under TCR or PI^35^.

IL-10, a well-studied example of an anti-inflammatory cytokine, appears in clusters associated with either monocyte or CD4+ T cell secretion under stimulation of those individual cell types (LPS or TCR, respectively) but is one of the few cytokines to be decreased due to communication under resting (cluster 9) or PI (cluster 14) stimulation. It is likely that other less well-studied cytokines in these clusters, such as CTACK (CCL27), may have an inhibitory role under some conditions.

### IP-10 and MIG are regulated synergistically in the mixture environment

**Figure 6:**
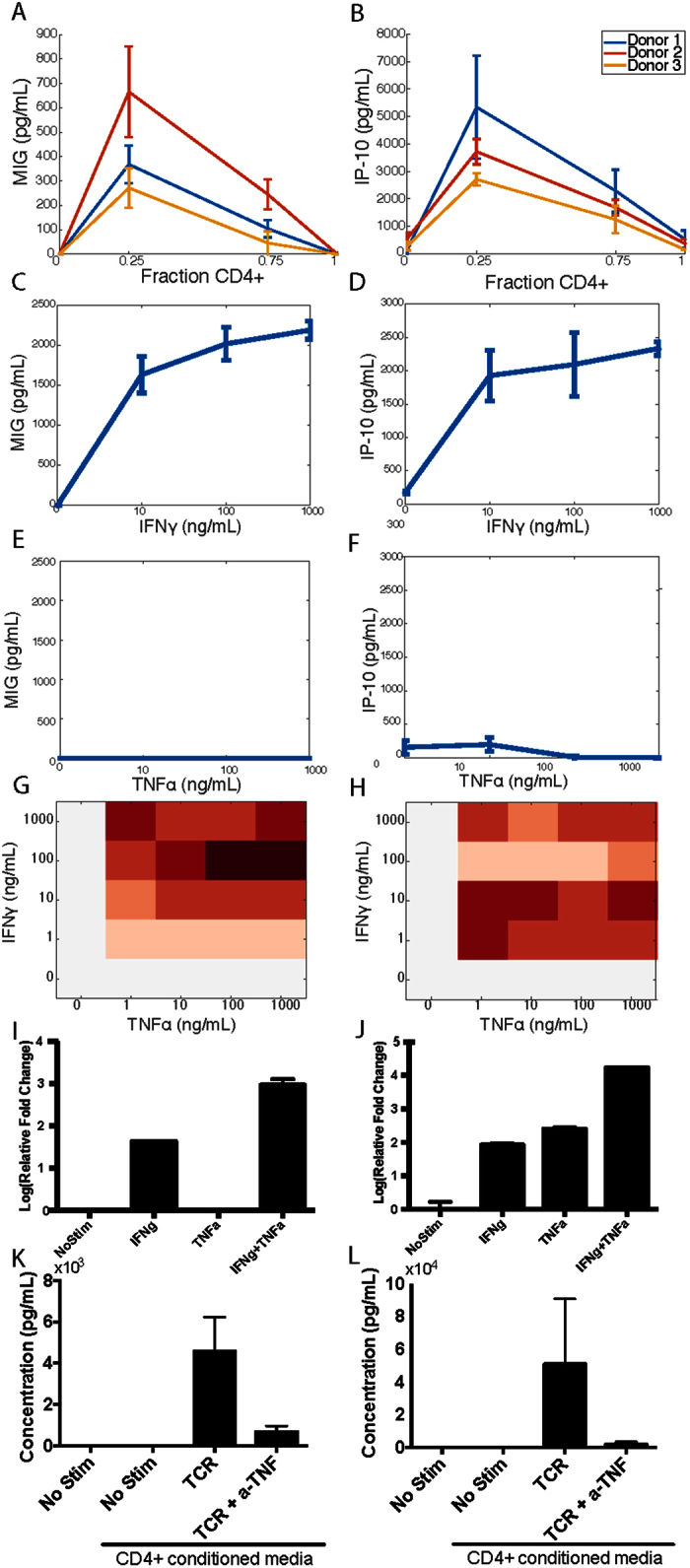
IP-10 and MIG are overrepresented in cocultures of CD4+ T cells and Monocytes. Data from our cocultures under TCR stimulus for MIG (**A**) and IP-10 (**B**) under increasing fractions of CD4+ T cells, where the remainder were monocytes from the same donor. Primary monocytes were stimulated with concentrations of IFNγ (**C,D**) or TNFα (**E,F**). Concentration of MIG (**C,E**) or IP-10 (**D,F**) was measured with a single-plex bead-based ELISA. (**G**) U937 cells were treated with combinations of TNFα and IFNγ as shown, and resulting MIG or (**H**) IP-10 was measured. Data were normalized to the maximum value of each cytokine observed. (**I**) mRNA from MIG or (**J**) IP-10 was measured at two hours with either no stimulus, 10 ng/mL TNFα, 10 ng/mL IFNγ, or 10 ng/mL each. Data were normalized by the ΔΔC_T_ method, with MIG data normalized to the no stim for IP-10, as no MIG mRNA was measured without stimulation or with TNFα stimulation. (**K**) MIG or (**L**) IP-10 concentrations were measured from supernatants of U937 cells unstimulated, or cultured with conditioned media from CD4+ T cells stimulated with TCR stimulus +/− 10 μg/mL of TNFα neutralizing antibody. All error bars represent mean ± standard deviation.

As one specific example of communication, IP-10 (IFNγ-inducible protein) was present at the highest levels in this dataset in the condition of 75% monocytes, 25% T cells when stimulated with TCR stimulus (Fig. 6B). IP-10 is a chemokine produced by monocytes among other cell types^36^. This is of particular note, as in our experiment, the stimulation of CD4+ T cells, which made up only ¼ of the cells present, caused a dramatic increase of secretion in monocytes. IP-10, along with I-TAC and MIG, binds to the CXCR3 receptor, and is secreted upon stimulation with interferons^37^. MIG was among the 48 cytokines measured, and upon further inspection, we see that MIG is also most highly secreted in the coculture conditions stimulated with TCR stimulus (Fig. 6A). In fact, MIG is not secreted in response to any of the other stimuli measured, nor is it present in isolated cell types in response to TCR stimulus.

IP-10 secretion can be induced by stimulation with IFNγ as well as in some cases type I IFNs or TNFα. As IFNγ and TNFα secretion were measured in our dataset, we observe that both are secreted in the conditions where IP-10 is detected (Supplementary Fig. S5). However, there is not good correlation between either IFNγ or TNFα levels and IP-10 levels, as CD4+ T cells alone also secrete IFNγ and TNFα in response to TCR stimulus. As secretion of IP-10 here appears to come from the monocytes, it is only in the mixtures that we see the highest levels of IP-10 AND MIG under TCR stimulation.

To determine whether secretion of IP-10 and MIG was regulated purely by IFNγ and monocyte number in the cocultures, we performed dose responses on primary monocytes with increasing concentrations of IFNγ (Fig. 6C,D). In our coculture model, we see responses as high as 5 ng/mL of IP-10 in the TCR condition. With recombinant protein, even doses as high as 1 ug/mL of IFNγ (approximately 200x higher than the highest observed IFNγ concentration in the coculture data) caused only 2.5 ng/mL of IP-10 secretion (Fig. 6D).

As previous studies have also reported control of IP-10 secretion by TNFα^38^,^39^, we additionally measured IP-10 and MIG secretion under TNFα stimulation, and found that TNFα does not appear on its own to cause secretion of IP-10 or MIG in primary monocytes(Fig. 6E,F). As we know both IFNγ and TNFα were present simultaneously in our coculture system, we treated U937 cells (a monocyte cell line) with combinations of IFNγ and TNFα. At every combination tested, we see synergistic secretion of both IP-10 and MIG, though the amount of synergy is differential between the two chemokines across combination concentrations. This synergistic secretion even with low levels (1 ng/mL) of IFNγ and TNFα may in part explain the high level of secretion observed in the coculture conditions (Fig. 6G,H).

To determine how synergistic regulation of IFNγ and TNFα may occur, we measured IP-10 and MIG mRNA in response to each treatment alone, or in combination. For IP-10, we find that both IFNγ and TNFα strongly upregulate mRNA, but the combination of the two is synergistic at the mRNA level (Fig. 6J). For MIG, we observe increased mRNA only with IFNγ treatment, and high synergistic mRNA levels with the combination treatment, despite no mRNA for MIG detected with TNFα alone (Fig. 6I).

Although we did not measure Type I IFN (IFNα and IFNβ) secretion here, previous work has also shown that Type I IFNs can regulate IP-10 and MIG secretion. We demonstrated that IFNα and IFNβ behave in a synergistic manner in combination with IFNγ (Supplementary Fig. S6). The regulation of IP-10 and MIG in response to TNFα, IFNγ, and other cytokines that may be present but were not measured here adds to the understanding of how IP-10 and MIG are regulated in a multicellular immune environment.

To confirm our proposed mechanism of synergistic IP-10 and MIG secretion, we treated U937 cells with conditioned media from CD4+ T cells treated with TNFα-neutralizing antibody. Neither IP-10 nor MIG were secreted from cells stimulated with conditioned media from resting CD4+ T cells. Secretion increased to expected levels in U937 cells stimulated with conditioned media from TCR stimulated CD4+ T cells - which suggests that secretion in the coculture is not primarily mediated through cell-cell contact between the cell types. U937 cells treated with conditioned media from CD4+ T cells stimulated with TCR stimulus and also with a TNFα-neutralizing antibody, showed reduced secretion of IP-10, I-TAC, and MIG compared to the condition without the neutralizing antibody, although not reduced to basal levels (Fig. 6J,K). This confirms that TNFα plays an important role in secretion of IP-10 and MIG in cocultures.

## Discussion and Conclusions

Here, we present a system of co-cultured immune cells, compared to their isolated cell counterparts, whole PBMCs, and plasma from the same human subjects. Across four culture conditions (three stimuli and resting), we observed the levels of 48 cytokines. In the case of two communicating immune cell types, the majority of cytokines we measured were altered from the expected value from measuring one isolated population under multiple stimulation conditions. This has broad implications for interpreting the multitude of data currently being gathered from PBMCs, as well as understanding cytokines present in serum and plasma – an even more complex environment in which cytokines are constantly diffusing into and out of tissues, degraded, or consumed.

The coculture data collected in this study combined with corresponding measurements from other physiological samples aides in interpretation of cytokine secretion data data collected from human plasma and PBMCs. As we have obtained corresponding measurements from multiple samples from the same donors, we are able to identify cytokines and conditions that are correlated and those that are not. We find that many more cytokines can be observed in cultured cell supernatants than are commonly detectable in plasma. We additionally find that secretion from the two isolated cell types studied is a poor predictor of PBMC secretion as compared to a simple two cell-type coculture. While cocultures between CD4+ T cells and monocytes improved prediction of PBMC secretion, they were not a perfect predictor, as many other cell types found in PBMCs can also secrete cytokines. These results suggest that communication between cell types is important in determining the overall cytokine environment.

Clustering using SOM allowed us to identify as key features of secretion patterns. For example, we could ascertain whether, under a given stimulation condition, a cytokine is secreted primarily by CD4+ T cells or by monocytes, and whether a cytokine is increased or decreased due to interaction between the two cell types. More cytokines were increased due to communication between cell types than decreased, as might be expected given the known ability of innate immune cells to activate adaptive immune cells, and increasing evidence that adaptive immune cells also influence the innate immune response^40^. As appropriate functioning of the immune system is dependent on coordination of many branches of a complex network of cell types and proteins, understanding this interplay between cells can potentially impact many studies of disease biology.

Finally, we utilize this method and analysis to uncover specific factors that might be contributing to the increased-over-expected secretion of IP-10 and MIG. By a combination of literature evidence as well as measurement of cytokine levels that are expressed in a similar set of communicating conditions, we uncovered a synergistic regulation of IP-10 and MIG by TNFα and IFNγ. The increased secretion of IP-10 and MIG in our system may be representative of the high levels commonly seen in inflammation and autoimmunity. As these two chemokines are targets of interest in clinical studies, a better understanding of their regulation can help interpret clinical results. Furthermore, as cytokines are increasingly targeted for interventions in autoimmunity, studies on regulation of individual cytokines affected by cell communication are necessary to understand the cytokine environment after a perturbation^41^.

Though the model system presented here only accounts for two cells, one could ultimately imagine a model comprised of infection-mimicking stimuli, cytokine stimuli, and many more cell types including tissue cells. Comparing cytokine concentrations measured in coculture to concentrations that would be predicted by a linear combination of cytokines from the two cell types allows for a systematic measure of the degree to which cytokines are affected by cell-cell interactions. This may ultimately inform choices of which cytokines are most amenable to perturbation in immune-mediated pathologies.

The physiology of both healthy and disease tissue is ultimately determined by the interplay of multiple cell types in complex extracellular environments. It has been difficult to date to precisely define circulating cytokines that represent disease states well. These experiments and subsequent analysis suggest a type of data that might be missing from the current studies of immune cell contributions to the cytokine environment. While the approach taken here represents only data from healthy subjects, the detection of many more cytokines and overall diversity of responses might ultimately lead to more insights into disease than can be gained from plasma alone. It is clear that this approach leads to findings about immune function and interactions between classically categorized innate and adaptive immune cells. Future work that includes more immune cell subsets and identifies disease-relevant immune cell communication will be useful in further clarifying the immune cell state in a variety of contexts.

## Experimental Procedures

### Cell isolation and stimulation

Peripheral blood mononuclear cells (PBMCs) were isolated using Lymphoprep (StemCell Technologies) from fresh whole blood from male human donors (Research Blood Components) collected in sodium heparin tubes. Sample collection was approved by the Research Blood Components IRB, and all methods were carried out in accordance with these guidelines. IRB approved informed consent was obtained for each donor. For each subject, one 10 mL vial was centrifuged for 15 minutes at 2000 g at 4 °C. Plasma was collected from the supernatant and frozen at −80 °C until use. PBMCs used for whole PBMC assays were frozen at 10 × 10^6^/mL in cryopreservation medium (90% FBS 10% DMSO). Monocytes and CD4+ T cells were isolated from fresh PBMCs using negative magnetic isolation (StemCell Technologies 19058 and 14052). Isolated cells were then frozen at 2 × 10^6^/mL in cryopreservation medium.

Cells were thawed and stained for CD4 and CD14 to validate CD4+ T cell and monocyte enrichment. Cells were incubated in PBS 1% BSA 0.1% tween-20 (PBS-TB) for 1 hour to block, then resuspended with FITC anti-CD4 (BD Pharmingen) and PerCP-Cy5.5 anti-CD14 (BD Pharmingen) and incubated overnight at 4C. The following day cells were washed 3x in PBS-TB and analyzed using an Accuri C6 flow cytometer (Becton Dickinson). Results were analyzed in FlowJo software (FlowJo).

Cells were cultured 24 hours in a 96 well U-bottom plate with cell compositions of 100,000 CD4+ T cells; 75,000 CD4+ T cells and 25,000 monocytes; 25,000 CD4+ T cells and 75,000 monocytes; or 100,000 monocytes. PMBCs were seeded at a density of 100,000 total cells per well. Mixtures of cells came from the same subject. Cells were unstimulated or stimulated with 2.5 μL/well anti-CD3/CD28 beads (TCR; Dynabeads, Thermo-Fisher) to stimulate T cells; 20 ng/well lipopolysaccharide (LPS, Sigma), to stimulate monocytes; or 1:500 diluted PMA/ionomycin cocktail (PI; Cell Stimulation Cocktail, eBioscience), to stimulate both cell types. Conditioned medium was collected at 24 hours post stimulation, clarified by centrifugation 15 min at 15,000 RPM and immediately used in Luminex assays. Each condition was performed in technical triplicate (3 wells for each stimulation condition) and biological triplicate (3 separate human donors). Cell culture was performed in RPMI media supplemented with 10% FBS.

Conditioned media experiments were performed by diluting supernatants exposed to stimulated or unstimulated CD4+ T cells for 24 hours 1:1 with fresh media, and exposing U937 cells for 24 hours. TNFα neutralizing antibody (D1B4) was purchased from cell signaling technologies and used at a concentration of 10 μg/ml.

U937 cells were purchased from ATCC and cultured according to manufacturer specifications.

### Viability Measurements

Viability was measured using cells from one subject after 24 hours of mono- or co-culture using propidium iodide (BioLegend). Cells and medium were collected after 24 hours of incubation, and wells were rinsed with PBS and rinsate collected to retrieve remaining cells from the bottom of the well. Propidium iodide was immediately added to cells at a 1:200 dilution. Cells were analyzed using an Accuri C6 flow cytometer (Becton Dickinson), and gating was performed manually.

### Cytokine Measurements

Cytokines in undiluted conditioned medium were measured by Luminex (BioPlex 27-plex and 21-plex cytokine kits) immediately following each experiment, without freezing. Protocols provided by the manufacturer were adapted to allow the assay to be performed in a 384 well plate to avoid introducing batch effects. Eight-point standard curves plus blanks (medium) were included for quantification and prepared according to manufacturers instructions.

For each cytokine, 5-parameter logistic curves were fit to the standards, including the blanks using MATLAB (MathWorks) and the L5P function (Cardillo G. (2012) Five parameters logistic regression http://www.mathworks.com/matlabcentral/fileexchange/38043). Curves were used to calculate concentrations for each sample replicate (Supplementary Fig. S7). Median fluorescence intensities for the samples below the lower asymptote or above the upper asymptote of the standard curve were imputed to be either the MFI of minimum asymptote or 99% of the MFI of the maximum asymptote, respectively. Lower limit of quantification was calculated for each cytokine as the lowest standard concentration that could be distinguished from (at least three standard deviations above) the media-only blank for that cytokine (Supplementary Table S1). One cytokine, IL-7 was excluded from further analyses because measurements for all subjects and conditions fell below the background, which was taken to be equal to the blank plus 3 standard deviations.

### Quantitative Real-Time PCR

U937 cells were seeded at a density of 500,000 cells/mL in 12 well plates and treated with 10 ng/mL of stimulus or media as shown for two hours. Total RNA was collected with the RNeasy Mini kit (Qiagen), and reverse transcription was performed with the QuantiTect Reverse transcription kit (Qiagen) according to manufacturers instructions. Bioinformatically validated primers were purchased from Qiagen, and SYBR green qPCR was performed with PowerUp SYBR green mastermix (Thermo Fisher) on a BioRad CFX384 real time system. Data were normalized according to ΔΔCT method based on a GAPDH control. All measurements were performed in technical triplicate and on at least two days. Representative data from one experiment are shown.

### Principal Components Analysis and Hierarchical Clustering

Technical replicates were averaged, and each signal was mean-centered and variance-scaled. PCA was performed using the pca function in MATLAB (MathWorks). Hierarchical clustering was performed using the clustergram function in the MATLAB Bioinformatics toolbox based on Euclidean distance between rows or columns.

### Self-Organizing Maps

Self-organizing maps were used to identify patterns of cytokine communication between CD4+ T cells and monocytes. Self-organizing maps allow for unsupervised clustering of data into lower-dimensional space, frequently selected to be 1- or 2-dimensions for visualization purposes.

Cytokine concentrations across different donors and stimuli were considered as a function of cell composition (100% monocytes, 75% monocytes with 25% CD4+ T cells; 25% monocytes with 75% CD4+ T cells; 100% CD4+ T cells) and were normalized to be between 0 and 1 across the 4 cell fractions. Cytokine-conditions that were zero across all 4 fractions were omitted. The SOM was generated using the MATLAB neural network toolbox (MathWorks, Natick, MA). The map was initialized with a 2-dimensional 4 × 4 square grid, resulting in 16 clusters, and the algorithm was allowed to continue for 10^7^ iterations, with an initial neighborhood size of 4. The number of clusters (2^4^) was selected to allow for clusters of high or low values for each of the 4 cell fractions, although varying the number of clusters did not affect the overall conclusions (data not shown).

## Additional Information

**How to cite this article**: Schrier, S. B. *et al*. Synergistic Communication between CD4+ T Cells and Monocytes Impacts the Cytokine Environment. *Sci. Rep.* **6**, 34942; doi: 10.1038/srep34942 (2016).

## Supplementary Material

Supplementary Information

## Figures and Tables

**Table 1:** R^2^ values of linear regression models of isolated cell types or mixture cell types used to predict PBMC secretion under each stimulation, across all three donors.

	LPS	PI	TCR	Rest
Isolated	0.80	0.79	0.45	0.71
Mixtures	0.94	0.77	0.50	0.86
