# Supplementary material for: Synergistic Communication between CD4+ T Cells and Monocytes Impacts the Cytokine Environment
Source: Sci Rep. 2016 Oct 10;6:34942. doi: 10.1038/srep34942 (PMC5056362; doi:10.1038/srep34942)
Supplement: Supplementary Information [file srep34942-s1.pdf]

Synergistic Communication between CD4+ T Cells and Monocytes

Impacts the Cytokine Environment

Sarah B. Schrier, Abby S. Hill, Deborah Plana, Douglas A. Lauffenburger*

Department of Biological Engineering, Massachusetts Institute of Technology, Cambridge, MA 02139, USA

Contact information: lauffen@mit.edu

Supplementary Information

	Lower Limit of Quantification (pg/ml)		Lower Limit of Quantification (pg/ml)
**MIP1b**	0.69	**IL17a**	28.17
**IL6**	9.20	**IL9**	25.04
**IFNg**	99.26	**IL2ra**	4.38
**IL1ra**	22.38	**MIG**	193.00
**IL5**	90.25	**IFNa2**	29.35
**GMCSF**	3.14	**SDF1a**	2.97
**TNFa**	253.14	**MCP3**	3.36
**rantes**	1.01	**IL16**	9.41
**IL2**	1.12	**IL12p40**	11.05
**IL1B**	8.41	**LIF**	1.86
**eotaxin**	104.85	**TNFb**	7.57
**FGFbasic**	62.68	**MIF**	67.96
**VEGF**	1.80	**IL18**	18.21
**PDGFbb**	96.57	**bNGF**	2.19
**IP10**	122.02	**GROa**	1.90
**IL13**	7.90	**HGF**	172.28
**IL4**	4.68	**IL1a**	5.16
**MCP1**	1.48	**IL3**	2.77
**IL8**	10.29	**SCF**	7.75
**MIP1a**	3.75	**TRAIL**	9.71
**IL10**	34.35	**MCSF**	25.09
**GCSF**	39.11	**CTACK**	3.57
**IL15**	21.80	**SCGFb**	113.64
**IL12p70**	36.80		

Supplementary Table S1: Lower limit of quantification for each cytokine.

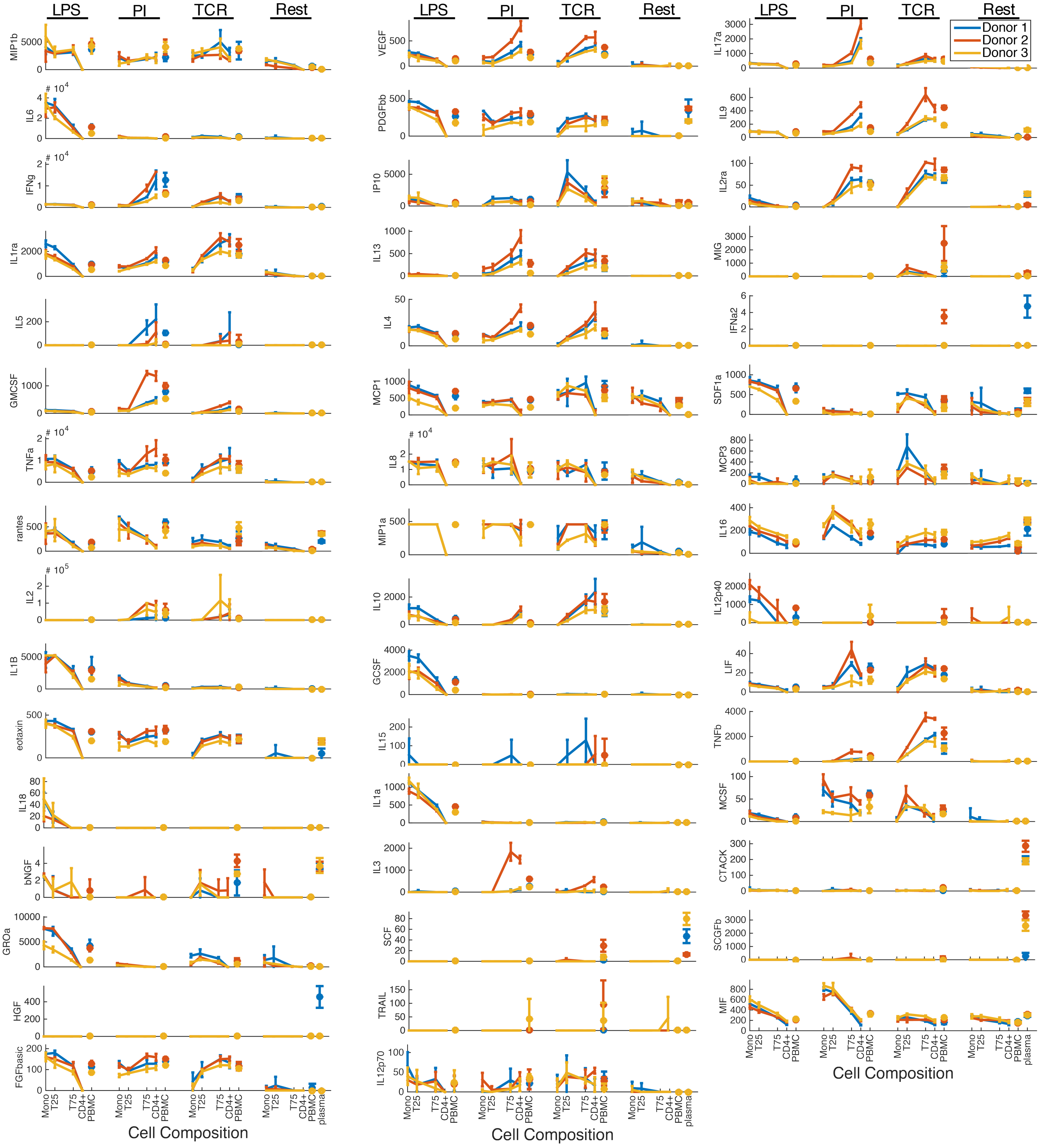

Figure S1: An overview of the collected data. Secreted or plasma concentrations of each cytokine across all of the stimulation conditions measured, for three donors, across each cell composition tested. Concentrations are measured in pg/mL. Each value plotted is mean ± standard deviation for technical triplicate measurements (with six replicates of plasma for each donor).

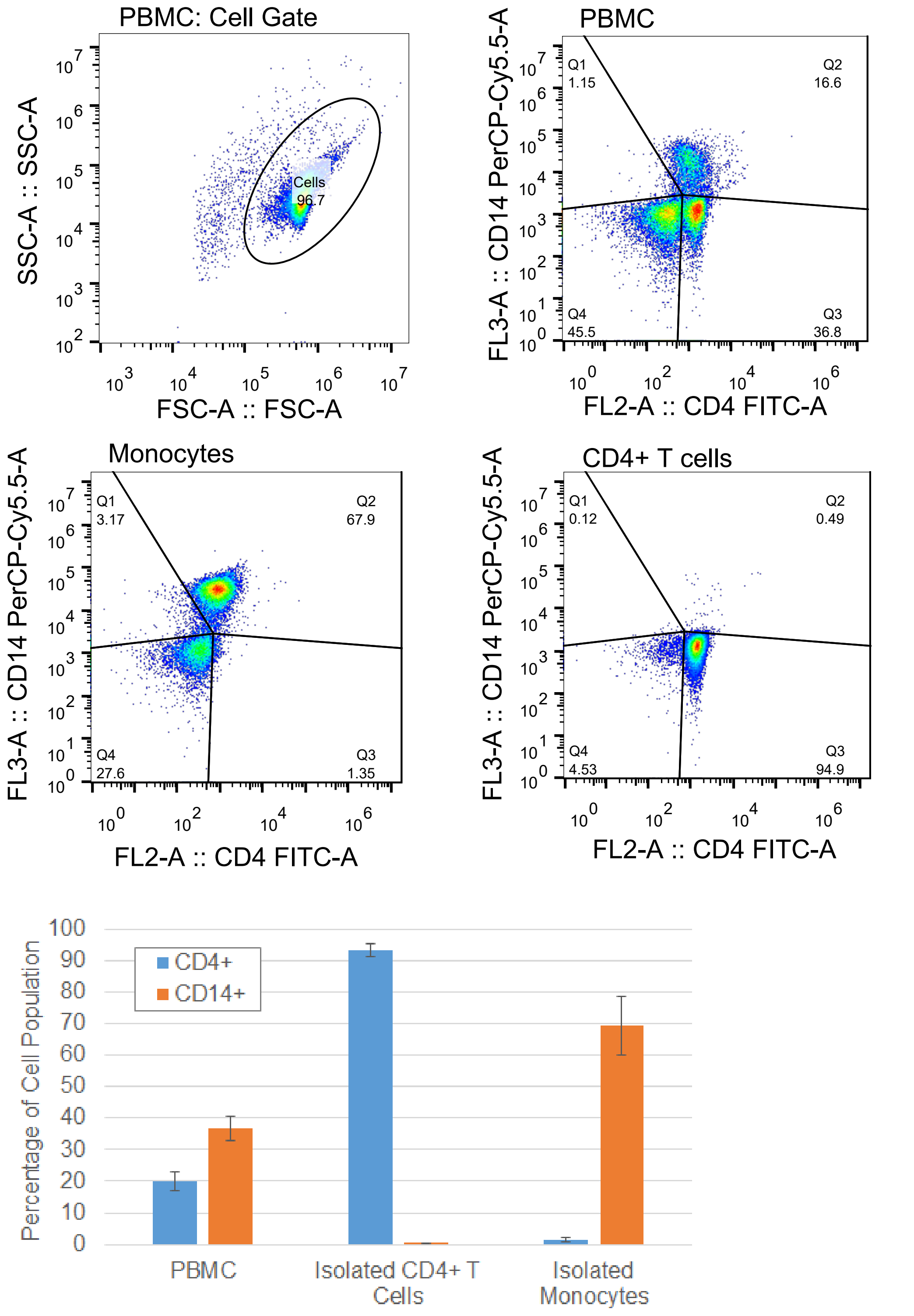

Supplementary Figure S2: Composition of isolated cell populations. PBMCs and isolated cell populations were stained for CD4 (CD4+ T cell marker) and CD14 (monocyte marker). Dot plots are representative results from one subject, and bar plot is mean +/- standard deviation across the three subjects.

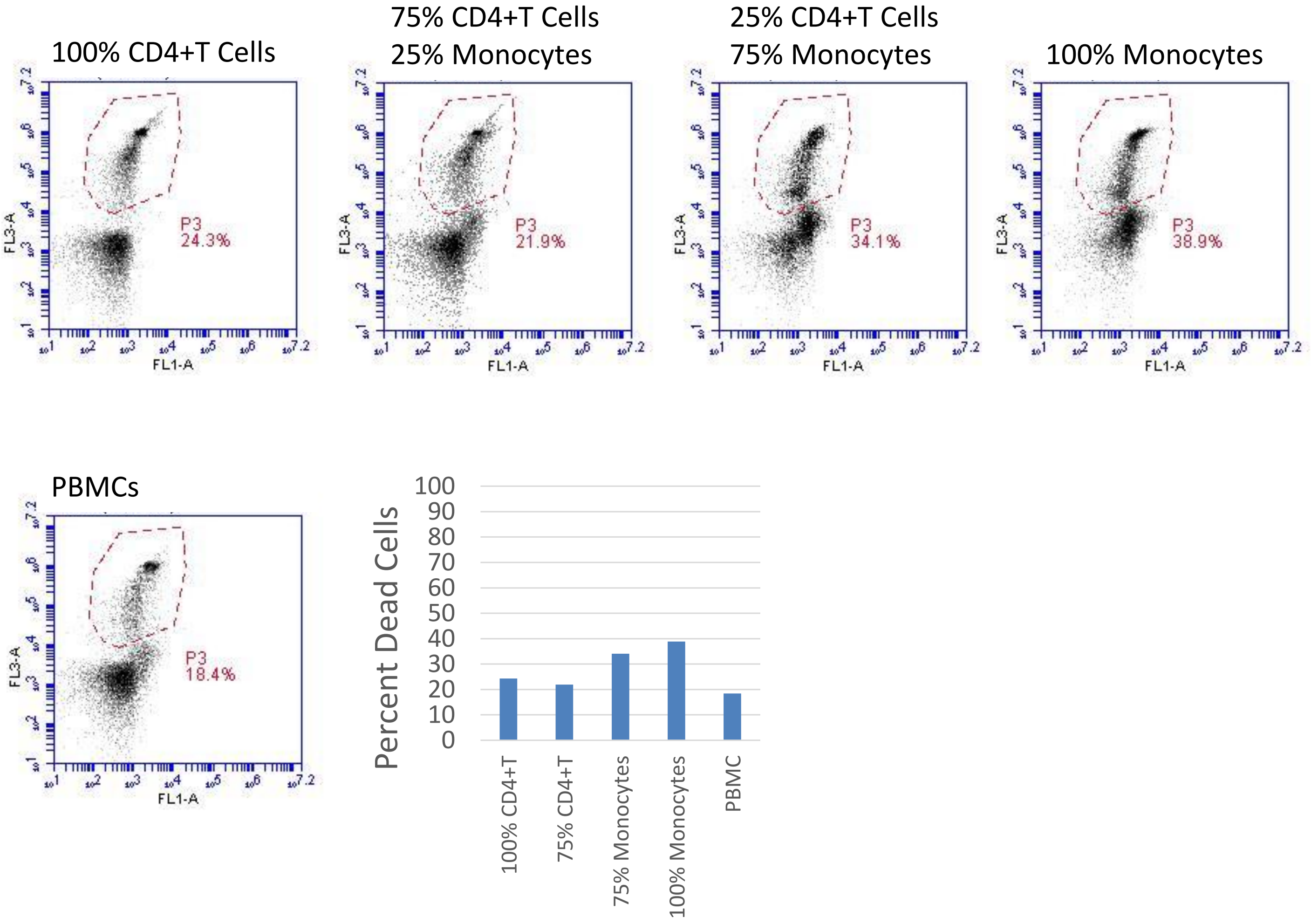

Supplementary Figure S3: Viability measured by propidium iodide. Viability is lower for monocytes after 24 hours, with no difference in viability observed due to interactions between cell types. Percentages shown are for dead cells.

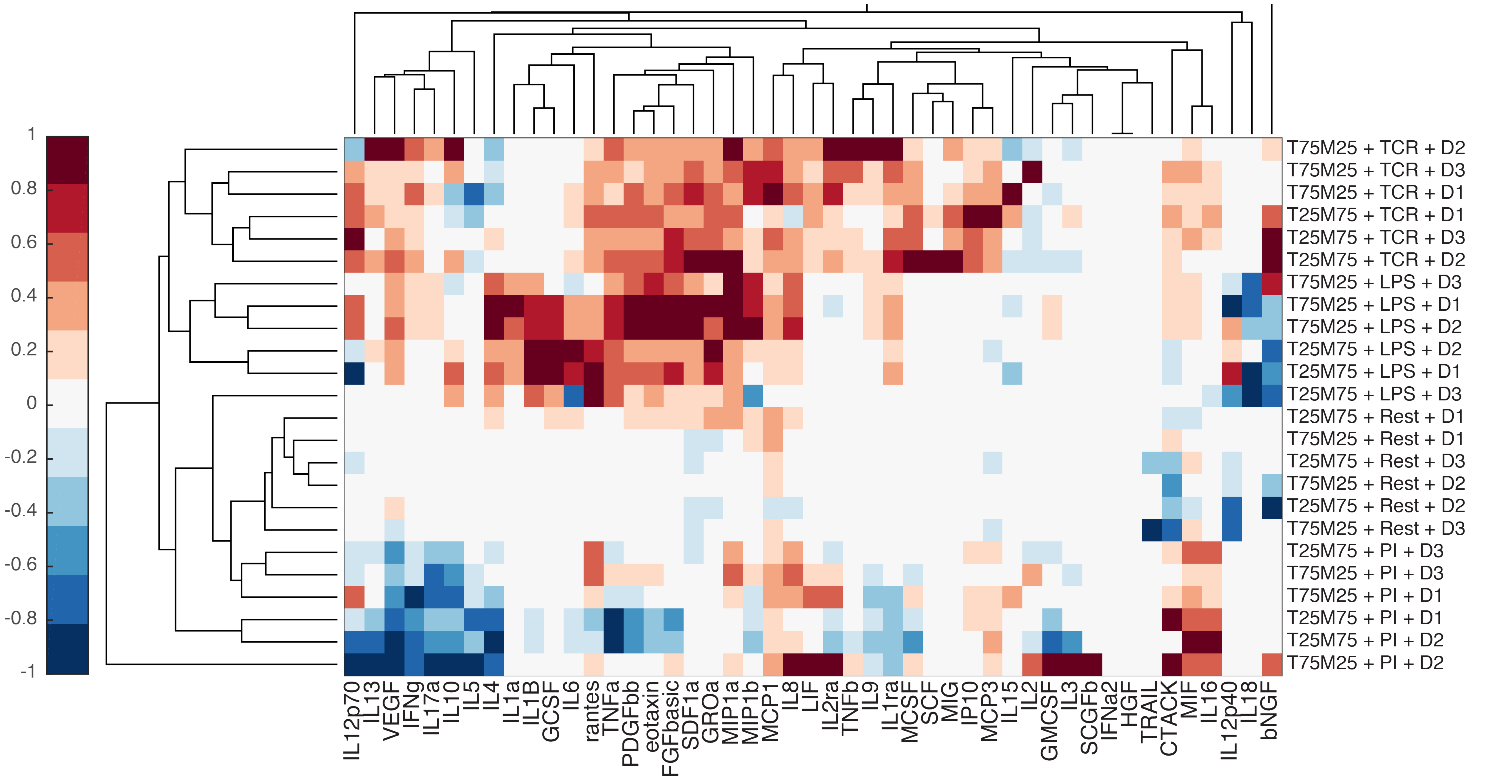

Figure S4: Cytokine levels are regulated differentially in a coculture environment. Differences between the expected and observed value for each cytokine under each condition, individually for all three donors, under four stimulation conditions are shown. Differences were normalized to the maximum difference for each cytokine.

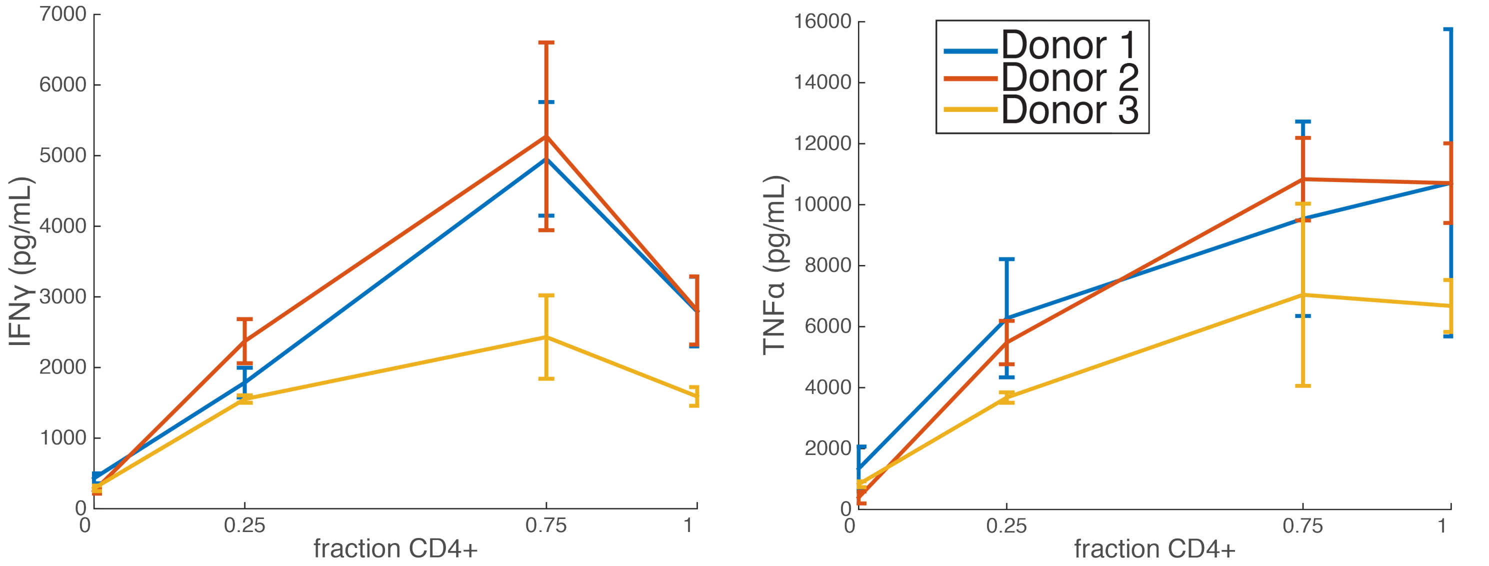

Figure S5: Coculture data of secretion of IFNγ and TNFα with TCR stimulus. Measurements displayed are mean ± standard deviation. X-axis represents cell composition of CD4+ T cells where the remainder was made up of monocytes from the same donor.

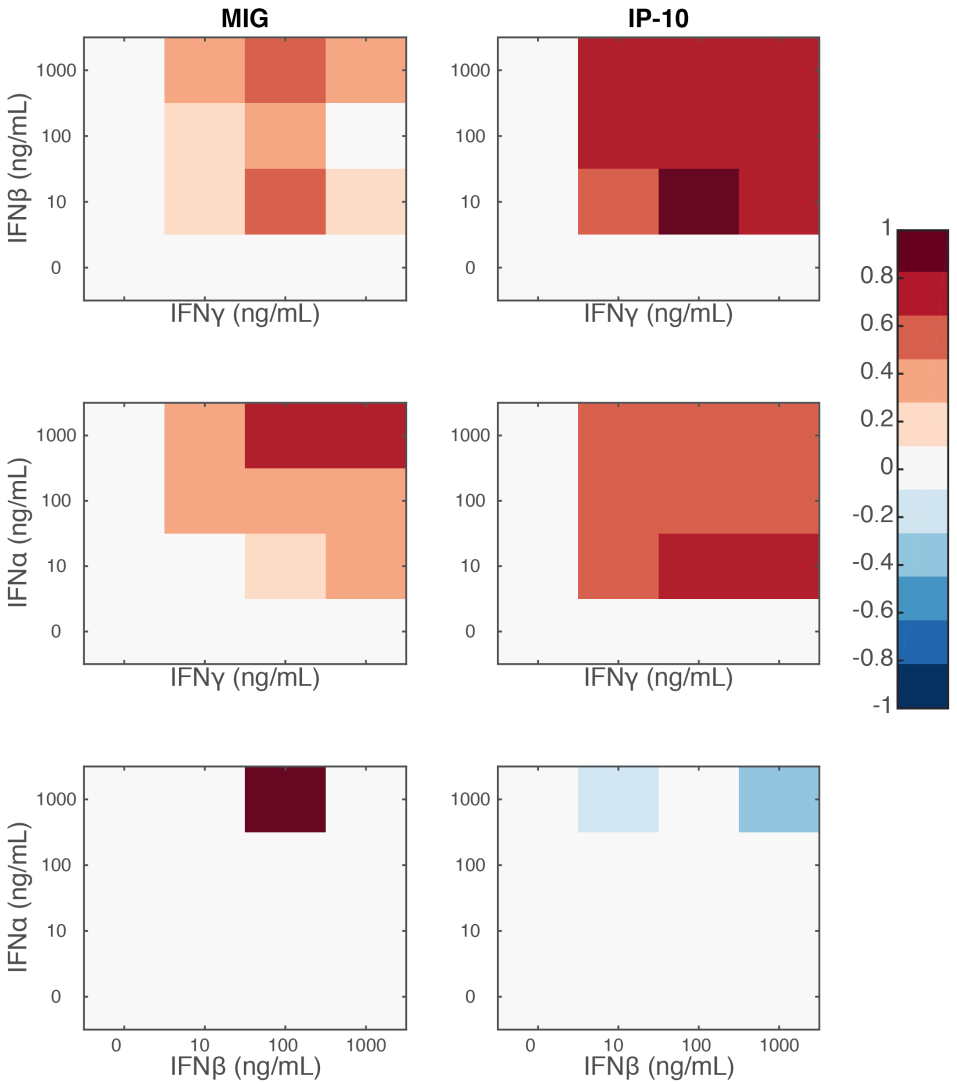

Figure S6: U937 cells were treated with combinations of IFNα, IFNβ and IFNγ as shown, and resulting MIG or IP-10 were measured. Data were normalized to the maximum value of each cytokine observed.

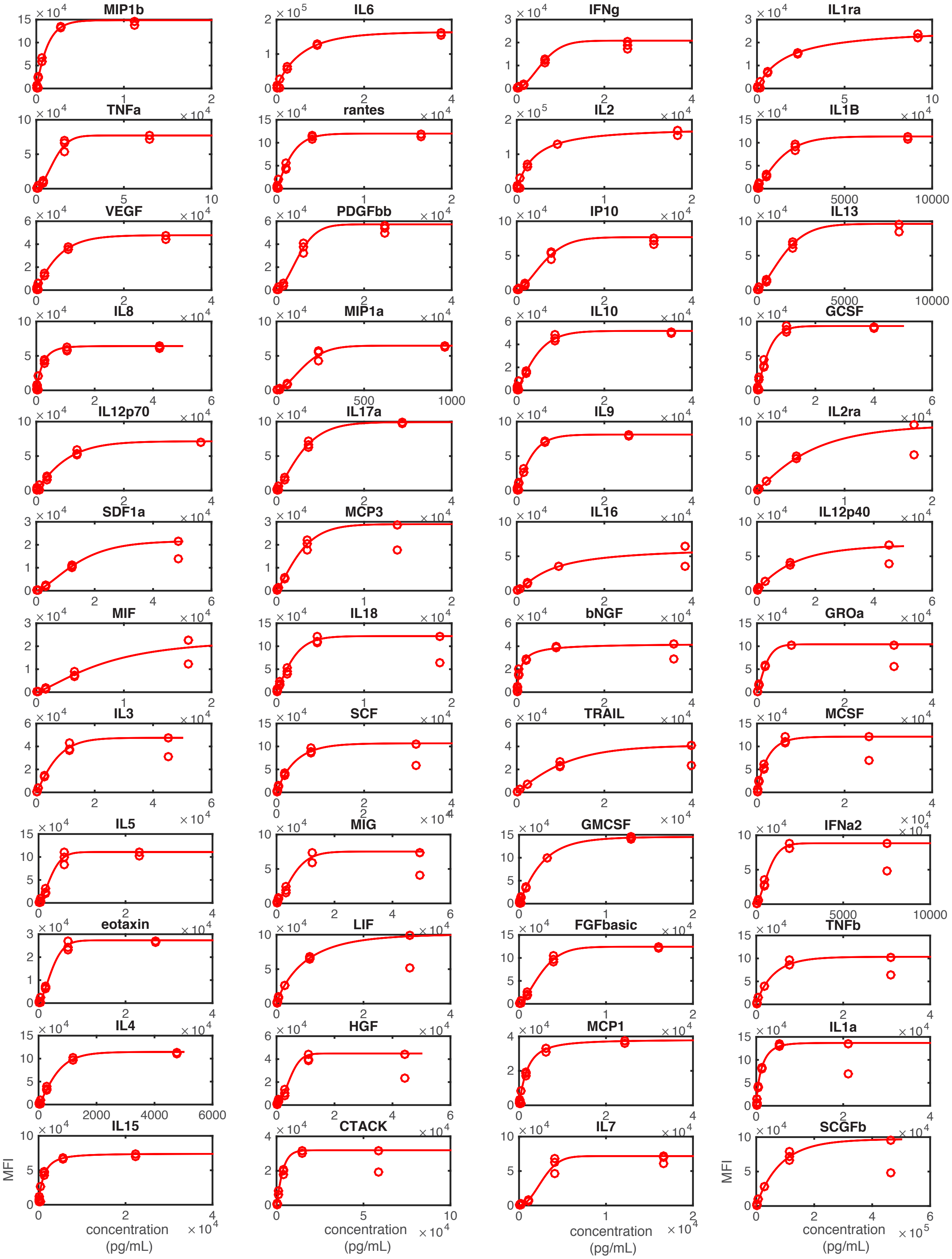

Figure S5: Standard curves for each cytokine measured. Triplicate of an 8-point standard curve with six replicates of a media-only blank are plotted by input concentration vs. measured mean fluorescence intensity (MFI). A five-parameter logistic curve was fit to each set of measurements, and used to calculate concentrations from measured data.
